# Case report: Dermatophytic pseudomycetoma in a domestic Korean short hair cat treated with intralesional injection of amphotericin B and oral terbinafine administration

**DOI:** 10.3389/fvets.2024.1402691

**Published:** 2024-06-13

**Authors:** Jaechun Cho, Chul Park, Jinho Park, Ji-Seon Yoon

**Affiliations:** ^1^Biosafety Research Institute and Laboratory of Veterinary Internal Medicine, College of Veterinary Medicine, Iksan, Republic of Korea; ^2^Biosafety Research Institute and Laboratory of Veterinary Dermatology, College of Veterinary Medicine, Jeonbuk National University, Iksan, Republic of Korea

**Keywords:** cat, dermatophytosis, amphotericin B, pseudomycetoma, intralesional injection, *Microsporum canis*

## Abstract

Dermatophytic pseudomycetoma (DPM), which is a deeper dermal and/or subcutaneous infection of dermatophytes, has been rarely reported in Domestic Korean Short Hair Cats. A 3-year-old, spayed female, domestic Korean Short Hair Cat presented with a history of crusts, nodules, and pruritus for 1 year. At the initial presentation, multifocal ulcerative nodules covered with yellowish grains were noted on her ventral thorax, abdomen, flank, and left hindlimb. Cytology of ulcerative nodules revealed degenerative neutrophils, macrophages, multinucleated giant cells, and hyphae. Histological examination of nodules revealed pyogranulomatous dermatitis with fungal plaques, and *Microsporum canis* and *Staphylococcus aureus* were identified in the culture. Therefore, the cat was diagnosed with DPM with secondary pyoderma. Oral itraconazole (10 mg/kg, once a day) was administered, but no significant improvement was observed. Therefore, intralesional (IL) injection of amphotericin B (0.6 mg/nodule) and oral administration of terbinafine (30 mg/kg, twice a day) were administered to the cat. With these medications, ulceration and the number and size of nodules decreased significantly, although large dome-shaped nodules remained. Skin lesions were treated with oral terbinafine and itraconazole administration for 5 months. However, after 6 months, recurrence of multifocal ulcerative nodules was observed, and the cat died 10 months after initial presentation. In this case, IL amphotericin B and oral terbinafine administration were partially effective in DPM treatment, suggesting that this may be an option for DPM treatment. Further studies to determine dose and frequency of IL amphotericin B in the management of DPM are warranted.

## 1 Introduction

Feline dermatophytic pseudomycetoma (DPM) comprises uncommon, deep dermal and sub-cutaneous fungal infections caused by dermatophytes, mostly *Microsporum canis* (1, 2). The majority of DPM infections have been described in Persian cats, suggesting a genetically programmed selective immunodeficiency (3). Clinical manifestations of DPM include nodular swelling, ulceration, draining sinuses, and grains in tissue that are micro-aggregates of the causative organism. Diagnosis of DPM is based on cytological and histopathological examination and microbiological culture (1–3).

There are limited reports regarding treatment for DPM. Treatment with griseofulvin, ketoconazole, terbinafine and itraconazole has shown varied response, and a progression of the disease was observed during the treatments (2, 4–6). Amphotericin B is a useful agent for the treatment of invasive fungal infections and is certified for the treatment of various fungal infections such as candidiasis and cryptococcosis in humans (7). The intralesional (IL) injection of amphotericin B induces high tissue concentration with few adverse systemic effects (8). In previous studies, IL injection of amphotericin B was reported to be effective for the treatment of nodular lesions caused by fungal infections such as sporotrichosis and protothecosis (9–12). However, very little information regarding the treatment of DPM in cats is available. In this case report, we describe DPM in a domestic Korean Short Hair Cat. Upon treatment with IL amphotericin B and oral terbinafine administration, the cat showed significant resolution of ulceration and nodules, while large nodular lesions remained and were less effectively treated.

## 2 Case description

A 3-year-old, spayed, weighed 3 kg, female domestic Korean Short Hair Cat living indoors presented with a history of nodular skin lesions, pruritus, and weight loss for 1 year. One year before hospitalization, the owner initially noticed skin lesions of crusts on the cat’s nasal bridge. After that, the skin lesions spread throughout the body and nodular lesions appeared. About 3 months before hospitalization, the cat was presented to the local animal hospital and diagnosed with dermatophytosis based on the results of fungal culture. After that, two injections of inactivated vaccine of *M. canis* (Biocan M, Bioveta, Czech Republic) and oral itraconazole (dose is not unknown) were administered. However, skin lesions were not resolved, and new multifocal nodular lesions appeared. The cat was living with another 13 cats, but clinical signs were only seen in this patient.

## 3 Diagnostic assessment, therapeutic interventions, and outcomes

**Figure 1:**
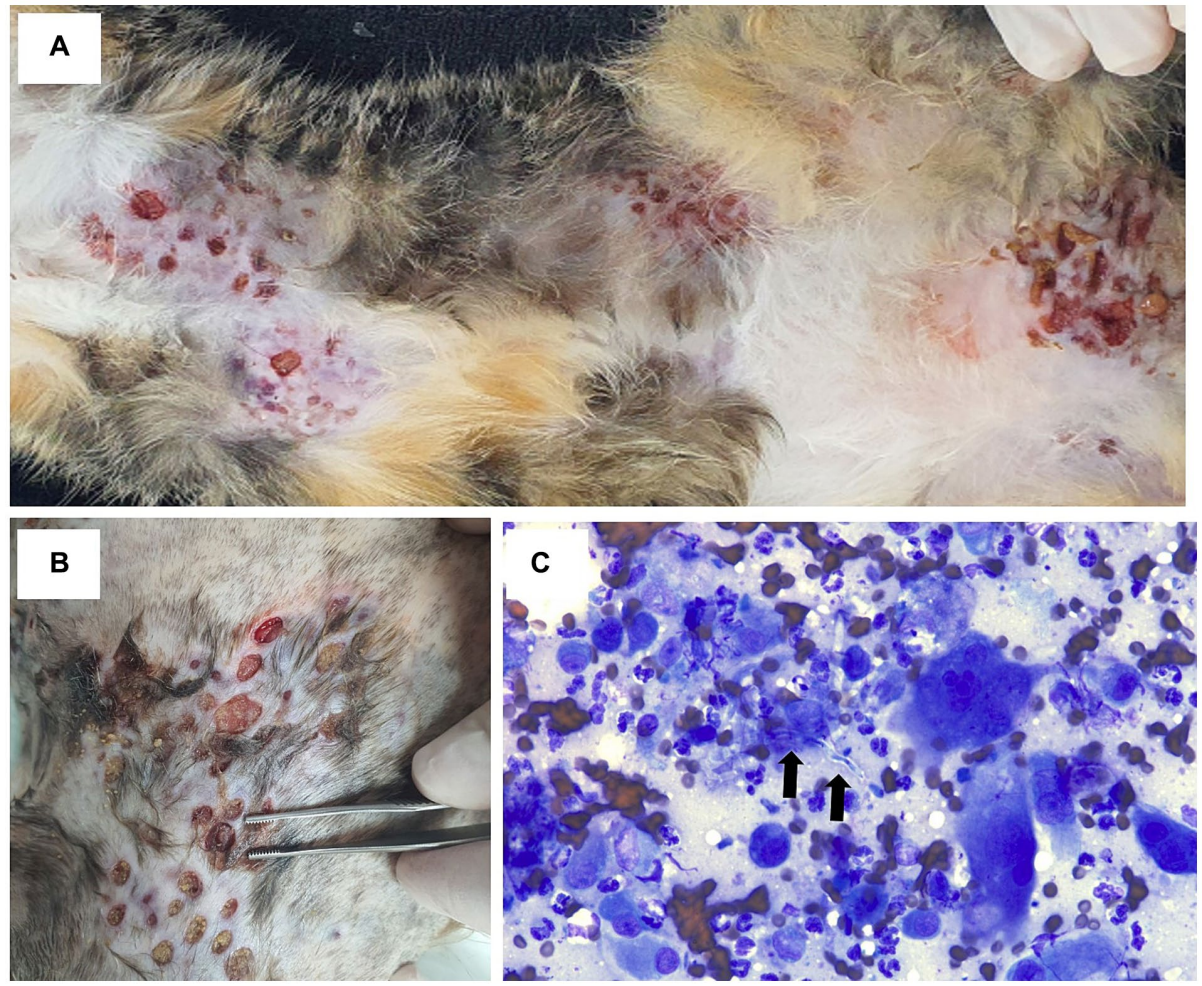
Clinical features and cytological examination of the present case. At the initial presentation, multifocal ulcerated nodules were observed on thorax, abdomen, and limbs. Some nodules showed serosanguinous discharge **(A)**. On the surface of ulcerative lesions, large numbers of yellowish grains were present **(B)**. In the cytology of ulcerative nodules, inflammatory cells including degenerative neutrophils, macrophages, and multinucleated giant cells, and hyphae indicative of fungal infection were detected **(C)**.

**Figure 2:**
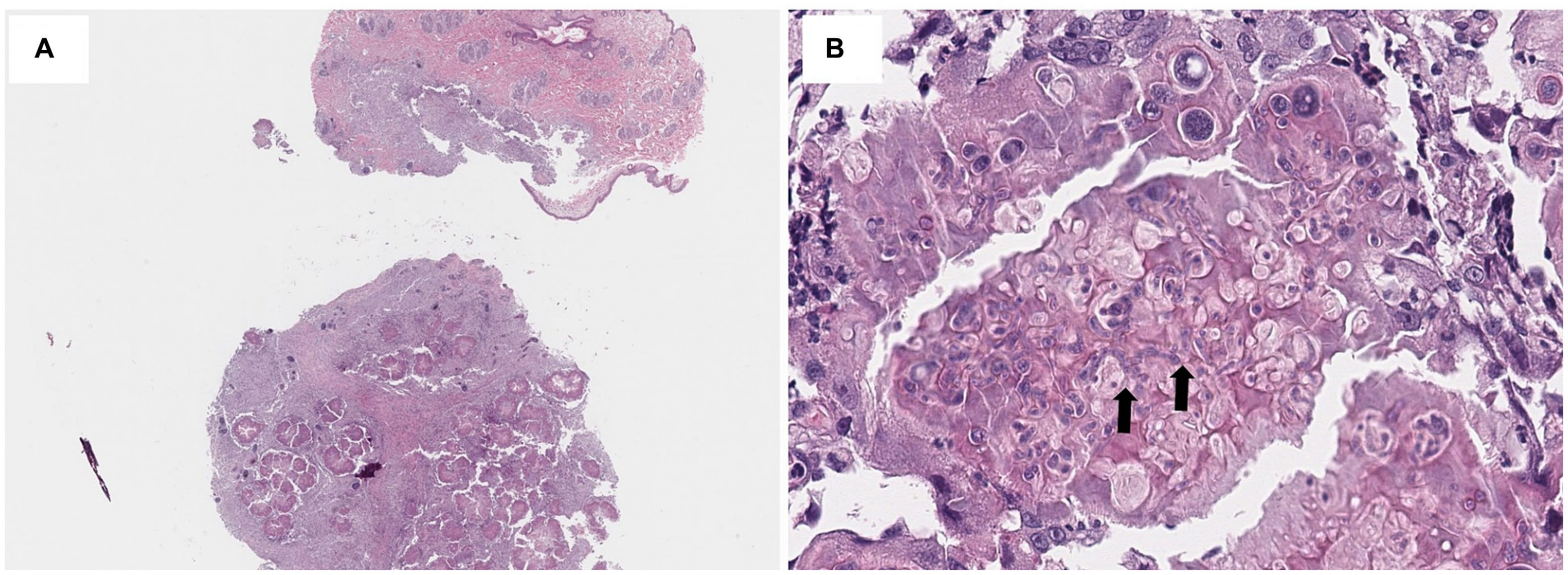
Histopathological findings of the present case. In histopathological findings, nodular infiltration of inflammatory cells comprising large numbers of macrophages and smaller numbers of neutrophils, lymphocytes, and plasma cells expanded the dermis and panniculus. Hematoxylin and eosin (H&E) staining, x40 **(A)**. The inflammatory infiltration is multifocally centered on irregular fungal organisms which include hyphae (arrow) with bulbous or spore-like dilatation. H&E staining, x400 **(B)**.

Upon initial presentation, multifocal ulcerative nodules were observed on the thorax, abdomen, flank, and limbs (Figure 1A). On the surface of the nodules, large numbers of yellowish grains were also noted (Figure 1B). In addition, in cytology of the ulcerative nodules, inflammatory cells mainly consisted of degenerative neutrophils, macrophages, and multi-nucleated giant cells were observed (Figure 1C). Furthermore, fungal hyphae were also noted upon fine needle aspiration of nodules (Figure 1C). Therefore, a deep fungal infection, such as DPM, was strongly suspected, and bacterial culture using blood agar and fungal culture using Sabouraud agar were performed (KVL Co Ltd., Seongnam, Korea). In addition, four punch biopsies (6 mm) were collected from the large nodule and subjected for histopathological examination (KVL Co Ltd., Seongnam, Korea). Colonies of aerobic cultures were identified as *Staphylococcus aureus* using matrix-assisted laser desorption ionization time-of-flight (MALDI-TOF) mass spectrometry. In addition, colonies of sabouraud agar confirmed *M. canis* through microscopic analysis. In histopathological analysis we found a severe inflammatory infiltrate that expands in the dermis, comprising large numbers of macrophages and fewer numbers of neutrophils, lymphocytes, and plasma cells (Figure 2A). The inflammation consisted of multifocally centered nodules of irregular fungal organisms which included irregular hyphae with bulbous or spore-like dilatation (Figure 2B). Therefore, based on the medical history, clinical signs, histopathological observations, and microbial culture, the cat was diagnosed with DPM caused by *M. canis* and secondary pyoderma. To investigate the systemic condition of the patient, we performed complete blood count and serum chemistry, which revealed leukocytosis (34.4; 10^9^/L; reference interval [RI]: 5.05–19.5), hypoalbuminemia (2.1 g/dL; RI: 2.3–3.5), increased ALT (112 U/L; RI: 22–84), increased AST (151 U/L; RI: 10–37), increased total bilirubin (0.8 mg/dL; RI: 0.1–0.4), increased amylase (2,125 U/L; RI: 200–1900), hyperglycemia (224 mg/dL; RI:71–148), hyperglobulinemia (5 g/dL, RI: 3.6–4.2), and increased feline serum amyloid A levels (21.3 μg/mL, RI: 0–5). In addition, the total T4 levels were within reference range and no positive reactions were observed using feline triple kit (Feline immunodeficiency virus; FIV, Feline leukemia virus; FeLV, Feline heartworm disease). In addition, feline anemia Real PCR with FeLV/FIV by ELISA were conducted (IDEXX Laboratories, Korea) and showed all negative tests (FeLV antigen by ELISA, for FIV antibody using ELISA and Real time PCR for *Cytauxzoon felis*, *Bartonella* spp.*, Anaplasma* spp.*, Ehrlichia* spp.*, Mycoplasma haemofelis, Candidatus Mycoplasma Haemominutum, Candidatus Mycoplasma turicensis*). Abdominal ultrasound and thoracic and abdominal radiographs were also unremarkable. Therefore, no underlying diseases were found as increased liver enzymes were thought to be due to the administration of anti-fungal agents and diabetes mellitus was ruled out as no hyperglycemia was observed upon re-examination.

**Figure 3:**
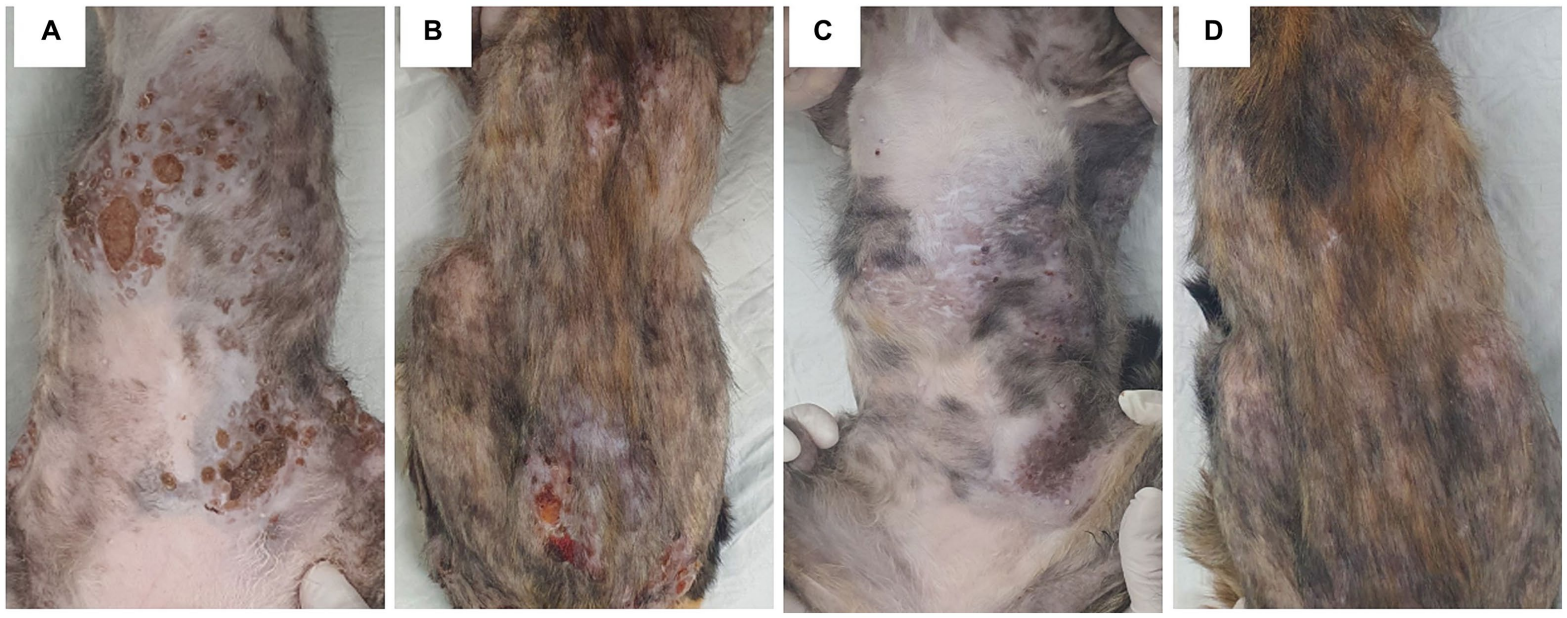
The changes in skin lesions after amphotericin B intralesional injection and oral terbinafine administration. Multiple and large ulcerative nodules were present on the thorax, abdomen, and trunk **(A,B)**. Upon three applications of amphotericin B intralesional injection and oral terbinafine administration, ulceration was significantly resolved, and the number and size of nodules were reduced. However, the size of large dome-shaped nodules remained unchanged **(C,D)**.

Treatments were started with oral administration of itraconazole (Sporanox; Janssen Korea, 10 mg/kg, once in a day) and cephalexin (Cefamedin; Ashish, 20 mg/kg, twice in a day), 0.05% chlorhexidine wipe and ketoconazole ointment (Nizoral cream, Janssen Korea). After 4 weeks of treatments, although pruritic levels were decreased, skin lesions of ulcerative nodules were not resolved, and new nodular skin lesions were observed on the abdomen (Figures 3A,B). We recommended surgical removal of large nodular lesions to the owner, but the owner refused to do it. Therefore, the decision to administer IL amphotericin B was made, as efficacy of IL amphotericin B in nodular lesions of other deep fungal infection has been reported. The cat was sedated with dexmedetomidine 20 mcg intramuscularly and 50 mg of amphotericin B (Ambisome, Gilead Sciences Inc) were diluted with 50 mL of normal saline 0.9% to obtain a final concentration of 1 mg/mL and IL injection amphotericin B (0.6 mg/lesion) were administered at 17 sites of nodular lesions. The total dose of amphotericin B per body weight was 3.4 mg/kg. After injection, the cat received IV infusion of normal saline for 2 h. Three applications of IL amphotericin B were performed at an interval of 1 week. In addition, as IL amphotericin B has been reported to be more effective in combination with oral antifungal agents, oral administration of terbinafine was also added. After 4 weeks of these medications, ulceration was significantly resolved, and the number and size of nodules were reduced (Figures 3C,D). However, large nodules did not show significant response to these drugs. After three applications of amphotericin B, mild elevation of BUN (42.5 mg/dL; RI: 17.6–32.8) was observed, but the level was normalized 2 months after cessation of amphotericin B administration. As 5 large nodules remained unchanged although oral terbinafine and itraconazole administrations were continued, further three applications of IL amphotericin B (0.6 mg/lesion) were conducted. However, no significant responses were observed. With the oral administration of terbinafine and itraconazole, skin lesions remained in that state. However, 9 months after initial presentation, multi-focal ulcerative nodules recurred and anemia (hematocrit:16.6%; RI: 30.3–52.3), leukocytosis (48.92 10^9^/L; RI: 5.05–19.5), increased ALT (392 U/L; RI: 22–84 U/L), and AST (384 U/L; RI: 10–37) liver enzymes, total bilirubin (0.6 mg/dL; RI: 0.1–0.4), and hyperglobulinemia (4.8 g/dL; RI: 3.6–4.2) were observed. Antifungal agents were administred, and supportive care, including fluid therapy, blood transfusion, and nasoesophageal tube placement were provided. However, the skin lesions and systemic condition were not resolved and the cat died 10 months after initial presentation.

## 4 Discussion

DPM has been rarely reported in domestic Short Hair Cats. Most cases of DPM have been reported in Persian cats which are predisposed to dermatophytosis and occasional cases of DPM (3, 5). In Persian cats, DPM can be formed through direct invasion of the dermatophytes to dermal tissues via inadvertent rupture of follicular structures (3, 5). Interestingly, in this case, although 13 cats were living together, clinical signs were only seen in this patient. As the owner stated that the lesions initially began with small crusts and hair loss on the nasal bridge, it was assumed that dermatophytosis first occurred in the superficial layer and then invaded to the dermal layer. Therefore, underlying systemic diseases that possibly induced DPM in this case were investigated but no significant abnormal findings were found.

In previous studies, a combination of surgery and long-term azole therapy were reported to be helpful in the management of DPM caused by *M. canis* in Persian cats (1). However, oral itraconazole monotherapy did not resolve the skin lesions in this case. In addition, the surgery could not be performed because the owner refused to do it. In previous reports, nodules caused by fungal infections of sporotrichosis and protothecosis were significantly resolved after treatment with IL amphotericin B (9–12). In this case of DPM, IL amphotericin B was partially effective for skin lesions as ulceration and some nodules were significantly resolved, but it was not effective for large nodules. In previous cases, IL amphotericin B was applied on localized nodules, but in this case, we applied it on multifocal nodules. We applied a lower dose per nodule than that reported in previous studies (9–11) to minimize the risk from increasing the total dose per body weight. The total dose per body weight (3.4 mg/kg) was higher than the dose administered by intravenous injection, as 17 sites were injected. Therefore, the reduced dose per nodule in this case may explain the relatively reduced efficacy of IL amphotericin B in DPM for this case. It may have been more effective to initially treat the larger nodules with the same doses as have been previously reported, rather than treating multiple nodules at once with a lower dosage. Further investigation of protocol details, including dose and frequency of IL amphotericin B administration in the management of DPM are required. In addition, when performing IL amphotericin B, caution should be taken to ensure that total dose relative to body weight is not increased.

In addition, combination therapy of IL amphotericin B with oral terbinafine was administered as previous reports suggested better efficacy of the combination versus that of amphotericin B alone (8). Terbinafine is an allylamine antifungal agent that suppresses the biosynthesis of ergosterol via inhibition of the fungal enzyme squalene epoxidase (12). In cats with DPM, successful resolution of DPM following terbinafine treatment were seen in two cats (5). However, one case report demonstrated a lack of response to oral terbinafine in DPM caused by *M. canis* in a Persian cat (6). In this report, after multifocal ulcerative lesions were largely resolved upon treatment with IL amphotericin B and oral terbinafine, skin lesions were maintained in the subsequent 5 months with oral administration of terbinafine and itraconazole. However, after 6 months when ulcerative multiple nodules recurred, those medications were not effective, and the cat died. Success in achieving complete resolution of DPM has rarely been reported. Relapses occur despite repetitive surgical excision combined with systemic antifungal therapy (4). This may imply that the infection extends beyond the visible borders of the lesion, and also indicates the invasion of deeper structures. In addition, immunodeficiency or aberrant immune responses caused by underlying diseases may be involved in the pathogenesis of DPM. Furthermore, in this case, large dome-shaped nodules were not resolved with the medications used, and it could be a possible source of further invasion of dermatophytes. Therefore, the prognosis may have been better if wide-marginal surgical removal and controlling underlying disease had been performed in this case.

Potential limitations in this study are as follows: the effects of single medications were difficult to evaluate due to the combination therapy. Further studies to compare the clinical effect of the combination therapy versus amphotericin B intralesional injection alone might provide further understanding for the efficacy of amphotericin B injection.

In conclusion, IL amphotericin B and oral terbinafine administration was partially effective in the treatment of DPM, suggesting that it may be an option for DPM treatment. Further studies to determine detailed protocol of IL amphotericin B in the management of DPM are required.

## Data availability statement

The original contributions presented in the study are included in the article/supplementary material, further inquiries can be directed to the corresponding author.

## Ethics statement

Ethical approval was not required for the studies involving animals in accordance with the local legislation and institutional requirements because as this manuscript is a case report, we obtained informed consent from the pet owners. Written informed consent was obtained from the owners of the animals for the publication of this case report.

## Author contributions

JC: Data curation, Investigation, Writing – original draft. CP: Data curation, Investigation, Writing – review & editing. JP: Investigation, Writing – review & editing. J-SY: Investigation, Writing – original draft, Writing – review & editing.
